# Real-world procedural and clinical outcomes of contemporary balloon-expandable and self-expandable TAVR systems: a single-center experience

**DOI:** 10.3389/fcvm.2025.1717839

**Published:** 2026-01-09

**Authors:** Mattia Vinciguerra, Andrea Spadafora, Alessandro Iaccarino, Fabio Miraldi, Eduardo Vitagliano, Antonio De Bellis, Ernesto Greco

**Affiliations:** 1 Department of Clinical, Internal, Anesthesiology and Cardiovascular Sciences, Umberto I Hospital, Sapienza University of Rome, Rome, Italy; 2 Department of Health and Life Sciences, European University of Rome, Rome, Italy; 3 Department of Cardiology and Cardiac Surgery, Casa di Cura “S. Michele”, Caserta, Italy

**Keywords:** balloon-expandable, paravalvular leaks, SAVR, self-expandable, TAVR

## Abstract

**Background:**

Transcatheter aortic valve replacement (TAVR) is rapidly becoming the preferred treatment for patients with severe aortic stenosis across different surgical risk profiles. However, the comparative performance of contemporary balloon-expandable valves (BEVs) and self-expandable valves (SEVs) in real-world practice remains debated.

**Objectives:**

To evaluate procedural and postoperative outcomes associated with different contemporary TAVR systems in a consecutive cohort of patients treated at a single center.

**Methods:**

We retrospectively analyzed 366 consecutive patients with symptomatic severe aortic stenosis and high surgical risk who underwent TAVR at Casa di Cura San Michele (Maddaloni, Italy) between January 2019 and October 2024. Patient baseline characteristics, procedural data, and in-hospital outcomes were collected and compared across different TAVR systems.

**Results:**

Among implanted prostheses, 71.0% were SEVs (Accurate Neo, Evolut R/Pro, Portico, Allegra) and 28.7% were BEVs (Sapien 3, Myval). BEVs were associated with significantly higher post-procedural transvalvular gradients (11.7 vs. 8.5 mmHg, *p* < 0.0001) but a lower incidence of moderate-to-severe paravalvular leak (2.9% vs. 6.9%). The need for permanent pacemaker implantation was more frequent in the BEV group (11.4% vs. 6.5%). Overall in-hospital mortality was 2.7%. Device success was achieved in 93.2% of procedures.

**Conclusion:**

In a real-world, single-center cohort, both BEVs and SEVs provided favorable outcomes. While BEVs demonstrated superior sealing properties with fewer paravalvular leaks, SEVs were associated with lower postoperative gradients. Careful consideration of anatomical and procedural factors remains essential for optimizing outcomes with contemporary TAVR systems.

## Introduction

The favorable risk-benefit profile has catalyzed the adoption of transcatheter aortic valve replacement (TAVR) from being considered a “last-resort” in prohibitive risk valve replacement to the treatment of severe aortic valve stenosis in high, intermediate and low risk surgery (1–4). Evidence from the randomized clinical trials has shown the superiority either the non-inferiority of TAVR vs. surgical aortic valve replacement (SAVR) with overall better composite endpoints including death, stroke and rehospitalization at 30-days (2–4).

The ongoing development of devices should be accounted for the successful factors of TAVR. Procedural safety and long-durability of the valve system are the leading reasons for technological refinement. In the last two decades, intra- or supra-annular valve design, self or balloon-expandable deployment system, potential for repositionability, hemodynamic performance and sheath size have been the key points of the available TAVR armamentarium.

The comparative effectiveness of contemporary devices is still a matter of debate with existing discordant findings from literature.

In this study, we sought to analyze the device-related complications and outcomes among a study population underwent the implantation of different TAVR systems.

## Materials and methods

### Study population

The retrospective consecutive registry from the Department of Cardiac Surgery of Clinica S. Michele (Maddaloni, CE, Italy), with symptomatic severe aortic stenosis and high-risk surgical profile between January 2019 and October 2024, underwent TAVR was analyzed. The study population received Myval (Meril Life Sciences Pvt. Ltd., Vapi, Gujarat, India), Sapien-3 (Edwards LifeSciences, Irvine, United States), or any of the following devices: Evolut R/PRO (Medtronic, United States), ACURATE neo (Boston Scientific, United States), Portico (Abbott, United States), and ALLEGRA (New Valve Technology AG, Switzerland). Clinical data were retrospectively gathered for all patients in a dedicated database after approval from the local ethics committee. In particular, we reported pre-, intra- and post-operative data of patients recruited, comparing them among the different TAVR systems implanted.

Transfemoral access was the preferred route in the vast majority of patients, while alternative percutaneous approaches were used only when femoral access was unsuitable. During the study period, all transapical procedures (4 out of 366, 1.1%) were performed exclusively with self-expandable (SEV) valves, according to institutional practice. Device selection was based on anatomical characteristics, operator judgment, and device availability.

### Statistical analysis

Continuous variables were expressed as means ± standard deviation and comparison between groups used the *t*-test. Categorical variables were compared using the Chisquare test. Statistical analyses were performed using Excel (Microsoft Corporation, Albuquerque, New Mexico, USA) and Stata 17.0 (StataCorp, College Station, TX, USA). A *p*-value <0.05 was considered to be statistically significant.

## Results

### Baseline characteristics

A total of 366 patients underwent TAVR between January 2019 and October 2024. The mean age was 81.95 years and 55.74% were males. Baseline clinical characteristics are summarized in Table 1. Systemic arterial hypertension was highly prevalent (94.54%), whereas diabetes mellitus type II affected 33.60% of patients (4.65% on insulin therapy). Peripheral arterial disease (28.96%) and chronic obstructive pulmonary disease (COPD) (28.96%) were equally represented. Neurological disorders were present in 12.84% of cases, and 1.91% had chronic kidney failure requiring dialysis.

**Table 1:** Pre-operative characteristics of the study population (*n* = 366).

Characteristic	Study population (*n* = 366)
Age, years	81.95 ± 6.32
Male gender, *n* (%)	204 (55.74)
BMI, kg/m^2^	29.17 ± 5.03
Arterial hypertension, *n* (%)	346 (94.54)
Diabetes mellitus type II, *n* (%)	114 (33.89)
Oral antidiabetic therapy	107 (29.24)
Insulin-dependent	17 (4.65)
NYHA functional class, *n* (%)	
I	22 (6.01)
II	145 (39.62)
III	178 (48.63)
IV	11 (3.01)
Peripheral arterial disease, *n* (%)	106 (28.96)
COPD, *n* (%)	106 (28.96)
Neurological disorder, *n* (%)	47 (12.84)
Chronic kidney failure—dialysis, *n* (%)	7 (1.91)
Laboratory tests	
Hemoglobin (g/dL)	12.43 ± 1.73
Creatinine (mg/dL)	1.17 ± 0.97
MDRD GFR (mL/min)	56.96 ± 23.65
Sinus rhythm, *n* (%)	267 (72.95)
Rhythm disturbance, *n* (%)	99 (27.05)
Permanent AF	54 (14.75)
Paroxysmal AF	20 (5.46)
Pacemaker	23 (6.28)
Sick sinus syndrome	1 (0.27)
Conduction block, *n* (%)	51 (13.93)
Incomplete LBBB	11 (3.01)
Complete LBBB	20 (5.46)
Complete RBBB	9 (2.45)
Incomplete LBBB + complete RBBB	11 (3.01)
AV node alteration, *n* (%)	29 (7.92)
AV block type I	23 (6.28)
AV block type II	3 (0.82)
AV block type III	3 (0.82)
Coronary artery disease	
Previous AMI and PCI, *n* (%)	47 (12.84)
Previous cardiac surgery	35 (9.56)
Echo findings: aortic valve	
Bicuspid valve, *n* (%)	9 (2.46)
Quadricuspid valve, *n* (%)	1 (0.27)
Peak aortic valve gradient (mmHg)	72.44 ± 22.64
Mean aortic valve gradient (mmHg)	44.74 ± 14.60
Aortic valve area (cm^2^)	0.76 ± 0.18
Echo findings: extra-valve involvement	
LVEF (%)	61.87 ± 9.59
LVESVi (mL/m2)	21.25 ± 16.10
SPAP (mmHg)	39.07 ± 13.72
TAPSE (mm)	22.07 ± 4.88
Moderate-to-severe AR, *n* (%)	19 (5.19)
Moderate-to-severe MR, *n* (%)	33 (9.02)
Moderate-to severe TR, *n* (%)	23 (6.28)

Regarding cardiac rhythm, 72.95% of patients were in sinus rhythm and 27.05% had atrial fibrillation. Conduction disturbances, left bundle branch block (LBBB) or right bundle branch block (RBBB) affected 16.94% of the population, while AV block was reported in 7.92%. A previous history of acute myocardial infarction (AMI) treated with percutaneous coronary intervention (PCI) was documented in 12.84% and 9.56% had prior cardiac surgery. Pre-procedural echocardiographic showed a bicuspid aortic valve in 2.46% and a quadricuspid valve in one patient (0.27%). Additional echocardiographic parameters, including left ventricular ejection fraction (LVEF) and systolic pulmonary artery pressure (SPAP) are shown in Table 1. Moderate-to severe aortic regurgitation (AR) was present in 5.19%, mitral regurgitation (MR) in 9.02% and tricuspid regurgitation (TR) in 6.28%.

### SEV vs. BEV—baseline differences

Baseline characteristics of patients treated with SEVs and BEVs are reported in Table 2. Patients receiving SEVs were significantly older than those treated with BEVs (82.41 vs. 80.89 years, *p* = 0.031). Conduction disturbances were more common in the BEV group (19.05% vs. 11.92%). In particular, the prevalence of pre-existing complete RBBB or LBBB was significantly higher among BEV patients (6.67% vs. 1.53%, *p* = 0.0106). Atrioventricular block was also more frequent in the BEV group (10.48% vs. 7.69%), although without reaching statistical significance. Other baseline clinical and echocardiographic parameters were comparable between groups.

**Table 2:** Pre-operative characteristics of BEV and SEV population.

Characteristic	SEV (*n* = 260)	BEV (*n* = 105)	*p* value
Age, years	82.41 ± 6.12	80.89 ± 6.70	0.0310**
Male gender, *n* (%)	147 (56.54)	56 (53.33)	0.6479
BMI, kg/m^2^	29.33 ± 5.25	28.77 ± 4.51	0.4053
Arterial hypertension, *n* (%)	247 (95)	98 (93.33)	0.7949
Diabetes mellitus type II, *n* (%)	93 (35.77)	31 (29.52)	0.3215
Oral antidiabetic therapy	74 (28.46)	24 (22.86)	0.1319
Insulin-dependent	19 (7.31)	7 (6.67)	0.8119
NYHA functional class, *n* (%)			
I	13 (5.)	9 (8.57)	0.2201
II	107 (41.15)	37 (35.24)	0.3763
III	124 (47.69)	54 (51.43)	0.7032
IV	10 (3.85)	1 (0.95)	0.1449
Peripheral arterial disease, *n* (%)	76 (29.23)	30 (28.57)	0.8668
COPD, *n* (%)	72 (27.69)	33 (31.43)	0.5899
Neurological disorder, *n* (%)	34 (13.08)	12 (11.43)	0.6589
Chronic kidney failure—dialysis, *n* (%)	3 (1.15)	4 (3.81)	0.1014
Laboratory tests			
Hemoglobin (g/dL)	12.25 ± 1.69	12.87 ± 1.77	0.0044**
Creatinine (mg/dL)	1.13 ± 0.86	1.26 ± 1.20	0.2698
MDRD GFR (mL/min)	57.04 ± 23.09	56.68 ± 25	0.9344
Sinus rhythm, *n* (%)	189 (72.69)	77 (73.33)	0.9731
Rhythm disturbance, *n* (%)	70 (26.92)	29 (27.62)	0.9559
Permanent AF	37 (14.23)	17 (16.19)	0.6928
Paroxysmal AF	14 (5.39)	6 (5.71)	0.9247
Pacemaker	17 (6.54)	6 (5.71)	0.7549
Sick sinus syndrome	1 (0.39)	0 (0)	0.5223
Conduction block, *n* (%)	31 (11.92)	20 (19.05)	0.0992
Incomplete LBBB	8 (3.08)	3 (2.86)	0.9128
Complete LBBB	11 (4.23)	9 (8.57)	0.1087
Complete RBBB	8 (3.08)	1 (0.95)	0.2419
Incomplete LBBB + Complete RBBB	4 (1.53)	7 (6.67)	0.0106**
AV block, *n* (%)	18 (6.92)	11 (10.48)	0.2916
AV block type I	13 (5)	10 (9.52)	0.1272
AV block type II	2 (0.77)	1 (0.96)	0.8699
AV block type III	3 (1.15)	0 (0)	0.2678
Coronary artery disease, *n* (%)	62 (23.85)	25 (23.81)	0.9499
Previous AMI and PCI	36 (13.85)	11 (10.48)	0.3946
Previous cardiac surgery, *n* (%)	27 (10.39)	8 (7.62)	0.4203
Echo findings: aortic valve			
Bicuspid valve, *n* (%)	5 (1.92)	4 (3.81)	0.3087
Quadricuspid valve, *n* (%)	0 (0)	1 (0.95)	0.1180
Peak aortic valve gradient (mmHg)	72.41 ± 20.52	72.49 ± 26.96	0.9809
Mean aortic valve gradient (mmHg)	44.56 ± 13.69	45.14 ± 16.55	0.7682
Aortic valve area (cm^2^/m^2^)	0.77 ± 0.17	0.74 ± 0.18	0.4622
Echo findings: extra-valve involvement			
LVEF (%)	61.48 ± 9.76	62.74 ± 9.19	0.3212
LVESVi (mL/m^2^)	21.22 ± 16.73	21.30 ± 15.02	0.9831
SPAP (mmHg)	39.34 ± 12.94	38.55 ± 15.25	0.7520
TAPSE (mm)	22.50 ± 4.94	21.13 ± 4.66	0.1239
Moderate-to-severe AR, *n* (%)	15 (5.77)	4 (3.81)	0.4430
Moderate-to-severe MR, *n* (%)	25 (9.62)	16 (15.24)	0.1590
Moderate-to severe TR, *n* (%)	14 (5.39)	9 (8.57)	0.2865

**Statistically significant *p*-values.

### Intra-procedural and post-procedural outcomes

Table 3 summarizes intra- and post-procedural characteristics of the study population. Concomitant PCI was performed in 2.19% of patients. The vast majority underwent transfemoral TAVR, while transapical access was required in four cases (1.09%). Valve-in-valve procedures accounted for 2.46% of the cohort. All access sites were closed percutaneously, with a vascular closure failure rate of 1.64%, and major vascular complications occurred in 2.73% of patients. One intra-operative death was recorded (0.27%).

**Table 3:** Intra- and post- operative characteristics of the study population (*n* = 366).

Intra-operative characteristic	Study population (*n* = 366)
Concomitant PCI, *n* (%)	8 (2.19)
Major vascular complication, *n* (%)	10 (2.73)
Access site percutaneous closure device failure, *n* (%)	6 (1.64)
Major bleeding, *n* (%)	7 (1.91)
Valve-in-valve procedure, *n* (%)	9 (2.46)
Transapical access, *n* (%)	4 (1.09)
Intraoperative death, *n* (%)	1 (0.27)
Device success, *n* (%)	341 (3.17)
Implantation of two devices	4 (1.09)
Significant PVL	21 (5.74)
Self-expandable prostheses, *n* (%)	260 (71.04)
Evolut R/Pro	78 (21.31)
Accurate neo	167 (45.63)
Portico	14 (3.83)
Allegra	1 (0.27)
Balloon-expandable prostheses, *n* (%)	105 (28.69)
Sapien-3	64 (17.49)
Myval	41 (11.20)
Post-operative characteristic	
AMI, *n* (%)	1 (0.27)
Stroke, *n* (%)	8 (2.19)
Disabling	6 (1.64)
Not disabling	2 (0.55)
AKI, *n* (%)	22 (6.02)
Mild	17 (4.65)
Moderate-to-severe	5 (1.37)
Pacemaker implantation, *n* (%)	29 (7.92)
New-onset atrial fibrillation, *n* (%)	26 (7.10)
Infectious disease, *n* (%)	10 (2.73)
UTI	4 (1.09)
Pneumonia	3 (0.82)
Sepsis	3 (0.82)
Thoracentesis or thoracostomy tube insertion	10 (2.73)
Post-operative echo findings	
Peak aortic prostheses gradient, mmHg	16.75 ± 8.03
Mean aortic prostheses gradient, mmHg	9.46 ± 4.95
Moderate-to-severe PVL, *n* (%)	21 (5.74)
Hospital stays, days	7.76 ± 4.80
In-hospital mortality, *n* (%)	10 (2.73)

Overall device success, according to Valve Academic Research Consortium-3 (VARC-3) criteria, was achieved in 93.17% of procedures. Moderate-to-severe paravalvular leak (PVL) occurred in 5.74% of cases, and a second valve was required in 1.09%.

Among all implanted prostheses, 71.04% were self-expanding valves (SEVs) and 28.69% were balloon-expandable valves (BEVs). SEVs included Acurate Neo (45.63%), Evolut R/Pro (21.31%), Portico (3.83%) and one Allegra case (0.27%). BEVs consisted of Sapien 3 (17.49%) and Myval (11.20%).

Post-procedural in-hospital mortality was 2.73%, and mean hospital stay was 7.76 days. The most frequent complications were permanent pacemaker implantation (PPI) (7.92%) and new-onset atrial fibrillation (AF) (7.10%), followed by VARC-3 acute kidney injury AKI (6.02%), moderate-to-severe PVL (5.74%), infectious complications (2.73%), and pleural effusions requiring drainage (2.73%). VARC-3 stroke occurred in 2.19% of patients, while one case of VARC-3 AMI was documented (0.27%). The mean post-TAVR gradient was 9.46 mmHg (Table 3).

### SEV vs. BEV—procedural and post-procedural differences

Table 4 presents intra- and post-procedural outcomes according to valve type. SEVs had a larger mean valve size compared with BEVs (26.22 vs. 25.00 mm). Hospital stay was significantly longer among SEV patients (8.12 vs. 6.90 days, *p* = 0.024). BEV recipients showed higher post-procedural gradients (11.72 vs. 8.47 mmHg, *p* < 0.0001).

**Table 4:** Intra- and post-operative characteristics of BEV and SEV population.

Intra-operative characteristic	SEV (*n* = 260)	BEV (*n* = 105)	*p* value
Concomitant PCI, *n* (%)	4 (1.54)	4 (3.81)	0.4207
Major vascular complication, *n* (%)	7 (2.69)	3 (2.86)	0.9467
Access site percutaneous closure device failure, *n* (%)	4 (1.54)	2 (1.91)	0.8168
Major bleeding, *n* (%)	4 (1.54)	3 (2.86)	0.4207
Valve-in-valve procedure, *n* (%)	4 (1.54)	5 (4.76)	0.0797
Transapical access, *n* (%)	4 (1.54)	0 (0)	0.2007
Intraoperative death, *n* (%)	1 (0.39)	0 (0)	0.5223
Device success, *n* (%)	239 (91.92)	101 (96.19)	0.1508
Self-expandable prostheses, *n* (%)	260 (100)	/	/
SEV size, mm	26.22 ± 3.23	/	/
Evolut, *n* (%)	78 (30)	/	/
Accurate, *n* (%)	167 (64.23)	/	/
Portico, *n* (%)	14 (5.39)	/	/
Allegra, *n* (%)	1 (0.39)		
Balloon-expandable prostheses, *n* (%)	/	105 (100)	/
BEV size, mm	/	25 ± 2.16	/
Sapien, *n* (%)	/	64 (60.95)	/
Myval, *n* (%)	/	41 (39.05)	/
Post-operative characteristic			
AMI, *n* (%)	0 (0)	1 (0.95)	0.1180
Stroke, *n* (%)	6 (2.31)	2 (1.91)	0.8012
Disabling	4 (1.54)	0 (0)	0.2007
Not disabling	2 (0.77)	2 (1.91)	0.3558
AKI, *n* (%)	14 (5.39)	8 (7.62)	0.4492
Mild	12 (4.62)	5 (4.76)	0.9731
Moderate-to-severe	2 (0.77)	3 (2.86)	0.1273
Pacemaker implantation, *n* (%)	17 (6.54)	12 (11.43)	0.1432
New-onset atrial fibrillation, *n* (%)	17 (6.54)	9 (8.57)	0.5312
Infectious disease, *n* (%)	6 (2.31)	4 (3.81)	0.4452
UTI	2 (0.77)	2 (1.91)	0.3558
Pneumonia	2 (0.77)	1 (0.95)	0.8699
Sepsis	2 (0.77)	1 (0.95)	0.8699
Thoracentesis or thoracostomy tube insertion, *n* (%)	7 (2.69)	3 (2.86)	0.9467
Post-operative echo findings			
Peak aortic prostheses gradient, mmHg	14.66 ± 6.52	20.95 ± 9.12	<0.0001**
Mean aortic prostheses gradient, mmHg	8.47 ± 4.59	11.72 ± 5.04	<0.0001**
Moderate-to-severe PVL, *n* (%)	18 (6.92)	3 (2.86)	0.1363
Hospital stays, days	8.12 ± 5.03	6.90 ± 4.07	0.0242**
In-hospital mortality, *n* (%)	6 (2.31)	4 (3.81)	0.4452

**Statistically significant *p*-values.

Moderate-to-severe PVL occurred more frequently in the SEV group (6.92% vs. 2.86%), although this difference did not reach statistical significance (*p* = 0.136).

Although not statistically significant, BEVs were associated with a higher incidence of PPI (11.43% vs. 6.54%), new-onset AF (8.57% vs. 6.54%), AKI (7.62% vs. 5.38%), and infectious complications. Conversely, stroke was slightly more frequent in SEV recipients (2.31% vs. 1.91%).

Valve-in-valve procedures were more common in the BEV group (4.76% vs. 1.54%), whereas all transapical procedures were performed with SEVs. Device success was slightly higher with BEVs (96.19% vs. 91.92%).

### Differential findings across the TAVR systems

**Figure 1:**
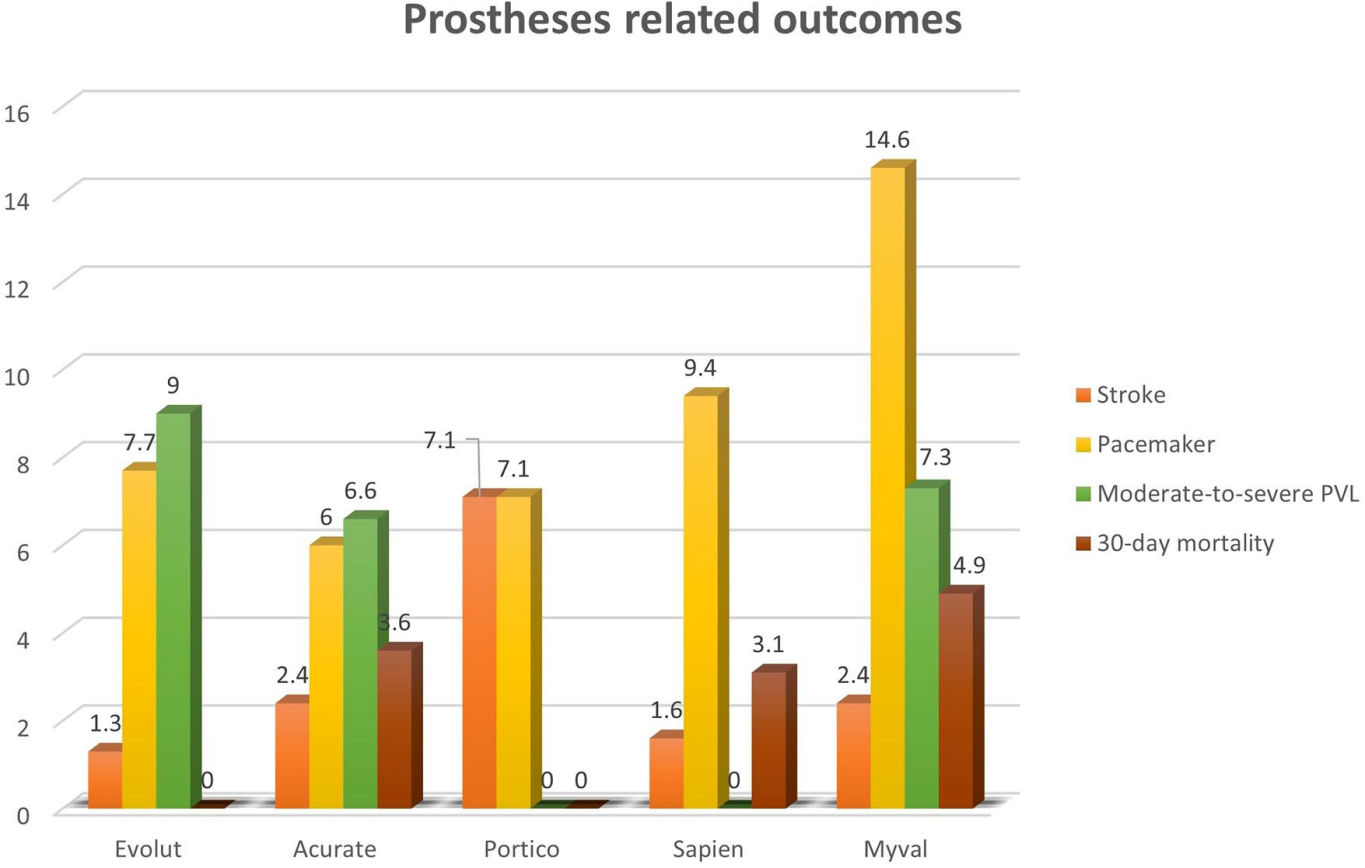
Related outcomes of the different BEV and SEV prostheses. Orange: stroke rate; blue: pacemaker implantation rate; green: moderate-to-severe PVL rate; brown: 30-day mortality.

The device-level distribution of post-procedural complications and in-hospital mortality is reported in Table 5 and illustrated in Figure 1. Stroke incidence remained below 2.5% for all valves except Portico (7.14%). PPI was highest with the Myval prosthesis, followed by Sapien 3, while SEVs showed rates between 6% and 8%. In-hospital mortality was numerically higher for Myval (4.88%), Acurate Neo (3.59%), and Sapien 3 (3.13%), although no differences reached statistical significance.

**Table 5:** Report of the related complications and mean aortic prostheses gradient of the different BEV and SEV prostheses.

Prosthesis	Stroke *n* (%)	Pacemaker implantation *n* (%)	Moderate-to severe PVL *n* (%)	In-hospital mortality *n* (%)
BEV (*n* = 105)				
Sapien 3 (*n* = 62)	1 (1.56)	6 (9.38)	0 (0)	2 (3.13)
Myval (*n* = 39)	1 (2.44)	6 (14.63)	3 (7.32)	2 (4.88)
SEV (*n* = 259)				
Evolut R/Pro (*n* = 78)	1 (1.28)	6 (7.69)	7 (8.97)	0 (0)
Acurate Neo (*n* = 163)	4 (2.40)	10 (5.99)	11 (6.59)	6 (3.59)
Portico (*n* = 14)	1 (7.14)	1 (7.14)	0 (0)	0 (0)
			*P* value	
Sapien-3 vs. Evolut R/Pro			0.0183**	
Sapien-3 vs. Acurate Neo			0.0408**	
Sapien-3 vs. Myval			0.02897**	

**Statistically significant *p*-values derived from chi-square tests comparing moderate-to-severe paravalvular leak rates among the different prosthesis.

Moderate-to-severe PVL occurred most frequently after implantation of the Evolut R/Pro valve (8.97%), followed by Acurate Neo (6.59%). Among BEVs, Myval showed a PVL rate of 7.32%. No significant PVL cases were recorded with Sapien 3 or Portico. Significant differences were observed when comparing Sapien 3 with Evolut R/Pro (*p* = 0.0183) and with Acurate Neo (*p* = 0.0408).

Mean post-procedural gradients are reported in Table 6. SEVs demonstrated consistent values around 8.5 mmHg. BEVs showed higher gradients, with Sapien 3 averaging 12.2 mmHg and Myval 11.08 mmHg (*p* < 0.0001 vs. SEVs). A mean gradient ≥20 mmHg was observed in 6 BEV cases vs. 4 SEV cases (*p* = 0.031).

**Table 6:** Mean aortic prostheses gradient among the different types of BEV and EV.

Group	Mean aortic prostheses gradient (mmHg)
Population study (*n* = 357)	9.46 ± 4.95
BEV (*n* = 105)	11.72 ± 5.04
Sapien (*n* = 64)	12.2 ± 5
Myval (*n* = 41)	11.08 ± 4.99
SEV (*n* = 259)	8.47 ± 4.59
Evolut (*n* = 78)	8.57 ± 5.45
Acurate (*n* = 163)	8.40 ± 4.15
Portico (*n* = 14)	8.74 ± 2.99
*p* value	
Balloon vs. self- expandable*	<0.0001*
Mean aortic prostheses gradient >20 mmHg	
SEV, *n* (%)	4 (1.54)
BEV, *n* (%)	6 (5.67)
*p* value	0.031*

*Statistically significant *p*-values.

## Discussion

TAVR is moving to become the treatment of choice for patients with aortic stenosis, demonstrating non-inferior outcomes rather than SAVR. The growing appeal for minimally invasive techniques with TAVR systems has led to a constant increase over SAVR (5). Transcatheter valves are categorized according to a balloon-expandable (BE) or a self-expandable (SE) concept. The accompanying balloon facilitates the expansion of BEV through its own radial strength. The deployment and expansion of SEVs happens automatically, being limited by the resistance of the annular wall.

Both designs are recommended indiscriminately in most clinical situations. The latest generations of devices have supplied some important limitations linked to initial TAVR devices, which included the inability of retrieval or repositioning after full expansion, large access sheath size and hemodynamic compromise during implantation. Stent designs have evolved to address the major complications (6). The choice of the TAVR to implant is influenced by specific anatomical characteristics and operators' confidence and expertise with the different valve delivery systems. Unfavorable anatomic factors orient toward a SEV.

In spite of the major technical differences, the type of prosthesis implanted may affect the survival benefit, making the decision of which valve to use a challenging task.

The current iterations of BEVs and SEVs have demonstrated excellent results in terms of the rate of the composite of death and rehospitalization early after surgery (4, 7).

The need for pacemakers, the rate of PVL and the incidence of stroke remain still open field of debate, with BEV prosthesis which have demonstrated better outcomes (8–10). We found significant shorter hospital stays for BEVs with an average of 6.9 days than 8.12 days of SEVs (*p* = 0.0242). The overall mortality rate was 2.73%, in absence of a statistical difference among the subgroups of prostheses implanted.

Furthermore, we focus on deeply analyzing such postoperative outcomes among the different new generation prostheses implanted.

### PMK implantation

The contact between the prosthetic valve and the left ventricular outflow tract is the primary cause of conduction disturbances after valve implantation. Damage of the AV node, the His and the infra-His system have been shown through electrophysiological studies (11, 12). The overall PMK implantation rate in the study population was 7.92%. BEVs were associated with an increased risk of PMK implantation, 11.43%, when compared to SEV, 6.54% (*p* = 0.1432) (Table 4). Such findings should be correlated to the different pre-operative characteristics of the two populations. Indeed, in Table 3 we report data on conduction abnormalities. A significant higher rate of incomplete LBBB and complete RBBB can be observed in the BEV group (6.67% vs. 1.53%, *p* = 0.0106).

The pre-existence of RBBB is a predictive factor for PMK implantation, increasing the risk of complete AV block after TAVR implantation (13–16). The left bundle branch can be damaged by the implantation of the prostheses due to compression or caused by periprocedural oedema of the left ventricular outflow tract (LVOT). This latter damage may be reversible, although there is no method to properly detect oedema after TAVR (17).

The magnitude and distribution of calcification affecting the aortic annulus wall and the landing zone contribute to being a predictive factor for PMK implantation after prostheses implantation (15). An asymmetrical expansion of the prostheses due to the calcium distribution may influence the mechanical stress on AV conduction system (17).

An additional factor may be related to the age of the population, with poor tissues in the elderly one (17).

Moreover, procedural factors associated with an overstretching of the LVOT may lead to an increased PMK implantation rate. Implantation depth into the LVOT is an independent risk factor with a direct correlation between the percentage of the prosthesis frame implanted into the ventricular side and the PMK implantation rate (18). Ballon valvuloplasty is related to exerting a damaging pressure on the AV node and His bundle (11, 19).

In our cohort, the numerically higher PMK rate observed with BEV could be partially explained by these anatomical and procedural mechanisms. BEV patients in our series presented a higher prevalence of baseline conduction disturbances (including incomplete LBBB and complete RBBB), known predictors of post-TAVR conduction block (13–16). In addition, procedural nuances such as implant depth or ballooning strategy were not systematically recorded and might have influenced the results. Consequently, the higher PMK rate with BEV in our study should be regarded as hypothesis-generating rather than causal. It should be noted that nearly half of the SEV cohort consisted of ACURATE Neo implants, a device characterized by lower radial force and consistently associated with lower PPI rates. This imbalance may have further contributed to the apparent inversion of the expected BEV–SEV PPI pattern.

In Table 5 and graphically in Figure 1, we report the rate of PPI of the different prosthesis implanted. Of note, Myval had the highest incidence of postoperative PPI, with 14.63%, followed by Sapien 3 (9.38%); Evolut and Portico showed a similar frequency around 7% and Acurate the lowest rate (5.99%).

The predictive factors afore mentioned may explain the wide range of PMK implantation rates after TAVI which exist in literature. In particular, for the Edwards Sapien 3 prostheses, the PMK implantation rate ranged from 4% to 24% (20, 21); for the Myval was around 19% (22); for the CoreValve Evolut R from 14.7% to 26.7% (23, 24); for the Portico prosthesis around 13.5% (25); for the Acurate Neo from 2.3% to 9.6% (26, 27).

In our series, the numerically higher PPI rate observed with the Myval prosthesis could reflect the early phase of device adoption at our center and differences in frame geometry and radial force distribution compared with other BEVs (22). Importantly, recent randomized evidence (COMPARE-TAVI 1) showed similar PPI rates between Myval and Sapien 3, supporting the interpretation that our finding likely reflects learning-curve and patient-selection factors rather than intrinsic device performance (22).

The lower risk of the Acurate Neo prosthesis may be associated with its specific design and deployment mechanism, resulting in lower radial forces and less pressure on the LVOT during expansion (26). The Evolut R/Pro prostheses are related to higher PPI rate in literature and it may be associated with the frame design responsible for a greater protrusion into the LVOT (27). For that reason, SEV and particularly Evolut prostheses are associated with greater PMK implantation rate than BEV. However, despite the procedural factors, as demonstrated by our results, pre-operative patients' characteristics are crucial in leading to a major susceptibility of AV node damage.

### Moderate-to-severe PVL

The major causes for post TAVR AR include the mismatch of the valve annulus and prosthesis diameter sizes, aortic root calcifications and a suboptimal device implantation. In our study, the SEV cohort included earlier-generation devices (Evolut R, ACURATE Neo, Portico), whose skirt designs differ substantially from the newer Evolut Pro/Pro+, ACURATE Neo2, and Navitor platforms. These updated designs have improved sealing skirts and are associated with lower PVL rates; therefore, the performance observed in our series should be interpreted in light of the specific generations represented. Short and long-term survival are negatively influenced by the development of a moderate-to-severe PVL (28). An adequate seal around the aortic annulus is critical to properly implanting the prosthesis. The presence of diffuse calcification on the aortic annulus and leaflets can hinder a tight sealing due to an asymmetrical expansion (29–32). In particular, the distribution of calcification on the aortic root, rather than the volume, may potentially determine PVL after TAVI. Cusp calcifications may lead to underexpansion, hindering a uniform contact between prosthesis and the landing zone (29, 32).

The aortic annulus sizes were found to be correlated with a major incidence of more than mild PVL, with smaller annulus size reported to be protective against the presence of PVL. Such findings have been explained through the better sealing of prosthesis and congruence on a small anulus. Of consequence, larger prosthesis, with a larger area to cover, may be associated with a higher risk of significant PVL (30). The other crucial factor for PVL development is prosthesis type. BEVs have demonstrated a better adaptation to the aortic valve annulus thanks to a higher radial force than SEV. An incomplete apposition of the SEVs stents to the annulus and LVOT is involved, further including anatomic variability which can influence the implantation such as an extreme angulation between LVOT and ascending aorta (29).

Moderate-to-severe PVL after TAVR affects the device success rate according to VARC criteria (33). In our series, the overall device success rate was 93.17%, including a moderate-to-severe PVL rate of 5.74% (Table 2). In Table 4 and Figure 1, we reported the incidence of more than mild PVL according to the prosthesis implanted. SEVs demonstrated a lower device success rate (91.92%) than BEVs (96.19%), with a higher moderate-to-severe PVL rate (6.92% for SEVs and 2.86% for BEVs, respectively). Table 5 shows the distribution of PVL rate among the different prosthesis implanted. In particular, the lowest incidence is associated with Sapien 3, with no cases reported, and the highest one with Evolut R/Pro (8.97%) with a significant difference between these first two (*p* = 0.0183) and Sapien 3 vs. Acurate Neo (6.59%, *p* = 0.0408). Similarly, among Portico prosthesis, no cases of moderate-to-severe PVL were observed; however, differently from Sapien 3 the small sample limited the statistical comparison. In the BEV group, Myval implantation was related to 7.32% of PVL cases, significantly higher than Sapien 3 (*p* = 0.02897).

**Figure 2:**
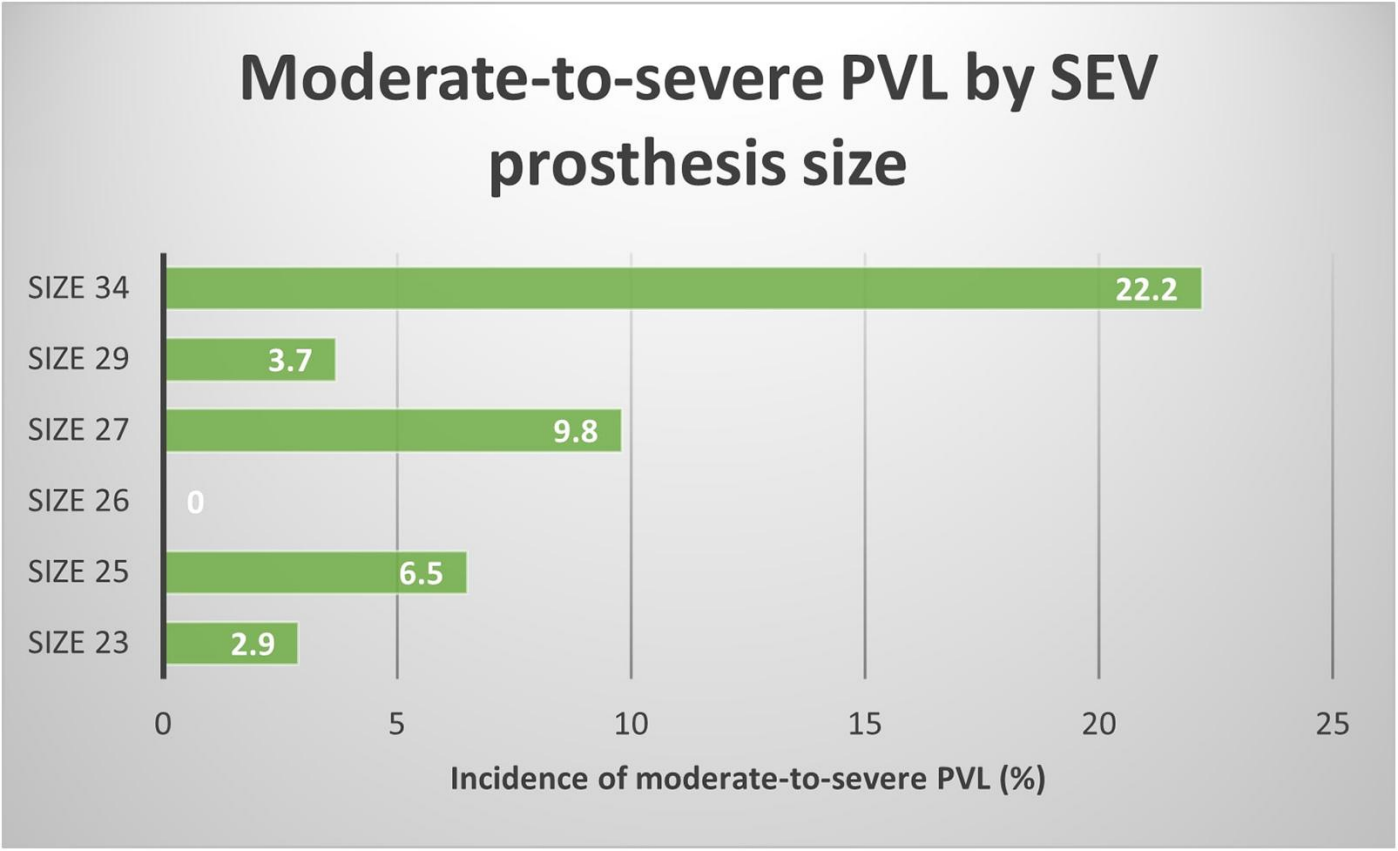
Association between moderate-to-severe PVL cases and the SEV prostheses size.

Figure 2 graphically reports the correlation between moderate-to-severe PVL rate and SEV prosthesis size. A growing trend of PVL incidence is directly correlated with prosthesis sizes. Such findings may confirm the hypothesis afore discussed regarding the protective role of smaller aortic annuli and prosthesis in causing PVL.

The contemporary generation of prosthesis have evolved to allow a better adaptation with aortic annulus, significantly reducing the incidence of PVL. Particularly, Sapien 3 and Evolut R/Pro, when compared to Sapien XT and CoreValve, respectively, have shown better procedural outcomes. They have been designed with the introduction of adaptive seal surrounding or by repositioning the valve in cases of suboptimal deployment (34, 35). Such implementations have led to reducing the impact of moderate-to-severe PVL, from 16.7% of CoreValve to 9% of Evolut R and more than 10% for Sapien XT up to 2.4% for Sapien 3 (34, 35).

The SCOPE 2 trial, comparing Acurate Neo and CoreValve documented the inferiority of the first against the latter prosthesis in terms of all-cause death or stroke at 1 year. The secondary analysis showed a higher PVL incidence at 30 days for the Acurate valve (10% vs. 3% of CoreValve, *p* = 0.002) (35). Portico prosthesis has evolved with large open cells design and an intra-annular deployment, ensuring a proper congruence with aortic annulus, demonstrating similar PVL rates when compared with the other commercially available valves (25, 36, 37).

Myval prosthesis offers more precise sizing through an extended matrix of available diameters. In our cohort, Myval was associated with a moderate-to-severe PVL rate of 7.32%, slightly higher than Sapien 3, which showed no cases of significant PVL. Nonetheless, through literature and our observations, when compared with Sapien 3, the latter has a better sealing against PVL (22). This observation may be influenced by specific anatomical factors, such as asymmetric or bulky annular calcifications, and by the different skirt configuration and sealing mechanism of the Myval valve. Despite this, recent randomized data from the COMPARE-TAVI 1 trial demonstrated comparable PVL rates between Myval and Sapien 3 (22), suggesting that the slightly higher PVL signal in our series likely reflects anatomical variability rather than intrinsic differences in device performance.

In general, the absence of standardized criteria and the great variability of imaging techniques used to detect PVL can explain the heterogeneity of range which exists in literature.

Our results confirm that Sapien 3 offers great adaptability to the aortic annulus morphology with excellent sealing results. On the other hand, the supra-annular design and self-expanding deployment, mainly in large aortic annuli, may be a factor predicting significant PVL development after the SEVs implantation.

### Stroke

Stroke is a life-threatening complication after TAVI, being associated with increased mortality and morbidity. Through a two-decades experience, the incidence of postoperative stroke has decreased from 6% in early reports up to 2% recently (2, 3, 23, 38, 45).

The safety and efficacy of TAVI have improved by new-generation devices. The relationship between thirty-day stroke and prosthesis type remains debated. An increased friction with the native aortic valve can be responsible for postoperative stroke, including as procedural triggering factors, larger devices, balloon expansion, the repositionable and retrievable mechanisms. The meta-analysis of Wang et al. (38) compares outcomes of SEVs vs. BEVs coming from randomized and propensity-matched studies. They concluded that stroke rates varied among the studies, founding a lowest incidence for Sapien BEV (1.90%) followed by Acurate SEV (2.60%) and Evolut SEV (3.30%), in absence of any statistical significance (38). They explained such variability through different diagnostic capabilities or the use of cerebral protection devices (CEP). These latter seem to be effective to reduce the risk of overall stroke and disabling stroke, although not decreasing the risk of non-disabling stroke (39).

Our examined study population showed an overall stroke rate of 2.19%, with 1.64% of disabling and 0.55% of non-disabling stroke, respectively (Table 2). The overall stroke rate is slightly higher for the SEV group (2.13% than 1.91% for the BEV group), in absence of statistical significance. In particular, the lowest stroke rate related to Evolut R/Pro implantation (1.28%), followed by Sapien 3 (1.56%), Acurate Neo (2.40%) and Myval (2.44%). One case of stroke was reported among Portico patients, showing an overall rate of 7.14%. However, this latter finding should be discussed in the context of the limited small sample size.

Cerebrovascular injuries may be caused by emboli released during the manipulation of atherosclerotic debris by delivery and deployment of TAVR systems. Patients and anatomic characteristics are crucial, together with the specific procedural factors, to predict in-hospital stroke. Among these, advanced age, alternative access, prior stroke or TIA, porcelain aorta and PAD were recognized as significant predictors (40).

Although the overall stroke rate of TAVI has reached the level of SAVR, important considerations are mandatory due to the identification of subclinical cerebral lesions in 68% of patients after TAVR. As more patients at intermediate or low-risk are considered for TAVR, it is essential to properly study the meaning of silent cerebral lesion in the context of a long life-expectancy (41).

### Others

The supra-annular position of the SEV leaflets can lead to a lower resistance to the LVOT, producing lower gradients than BEVs. The higher radial strength, in case of Sapien 3 implantation, ensures excellent congruence with the aortic annulus but at the same time is responsible for higher gradients (42, 43). Both peak and mean aortic prosthesis gradients are significantly higher in the BEV group than in the SEV, with 11.72 vs. 8.47 mmHg on average, respectively (values referred to the mean aortic prostheses gradients, *p* < 0.0001) (Table 4). As reported by Didier et al. (44) the rise in the mean gradient early after the procedure is not directly related to worse outcomes. However, if the gradient remains elevated, more than 20 mmHg, at 1-year follow-up it is associated with a worse 4-year survival rate. Their results suggest that two-thirds of patients with post-procedural means gradient more than that threshold, at 1-year reduce significantly such value. In Table 6, we report the mean aortic gradient for the different types of prosthesis, highlighting Sapien-3 and Myval had significantly higher values. Furthermore, we analyzed, between the two groups, patients with a mean gradient of more than 20 mmHg. Of note, among BEV patients, 5.67% of them showed such gradient against 1.54% of SEV population, demonstrating a significant difference in terms of hemodynamic performance (*p* = 0.031). Long-term randomized trial results regarding new-generation devices are missing and the potential structural valve deterioration related to such hemodynamic values remains unknown.

Notably, BEVs were preferentially selected for valve-in-valve (ViV) procedures in our cohort. This procedural choice likely contributed to the slightly higher post-procedural gradients observed in this subgroup. In ViV-TAVR, a mean gradient around 20 mmHg is generally considered acceptable, especially when associated with substantial pre-procedural gradient reduction and symptomatic improvement. Nevertheless, these values remain higher than those observed after native TAVR and should be interpreted in light of anatomical and procedural selection rather than intrinsic device performance (42–44).

Among the other major complications, we report a similar rate in terms of major vascular complication, peri-procedural AMI, potentially associated with coronary arteries occlusion, AKI and new-onset atrial fibrillation.

## Conclusion

In this real-world, single-center experience, new-generation balloon-expandable and self-expandable TAVR systems demonstrated overall favorable outcomes, with distinct procedural and hemodynamic profiles. BEVs offered superior sealing properties with lower rates of paravalvular leak, while SEVs were associated with lower postoperative transvalvular gradients. However, given the retrospective, non-randomized design and the limited sample size for some prostheses, these findings should be considered exploratory and hypothesis-generating. Future prospective, multicenter studies with longer follow-up are warranted to confirm these observations and to guide optimal device selection in clinical practice.

## Study limitations

This study has several limitations. First, its retrospective and observational design is subject to inherent biases, including potential residual confounding despite careful data collection. Second, the analysis was conducted at a single center, which may limit the generalizability of the findings to other institutions with different patient populations, operators' experience, and device selection strategies. Third, the study population was heterogeneous and not randomized, with unequal distribution of patients receiving balloon-expandable vs. self-expandable valves. Fourth, follow-up was restricted to the in-hospital phase, and therefore mid- and long-term outcomes, including structural valve deterioration and survival, were not evaluated. Moreover, nearly half of the SEV cohort consisted of ACURATE Neo valves, a device that did not achieve non-inferiority compared with contemporary SEV and BEV systems in the ACURATE IDE trial. This overrepresentation may have influenced comparative outcomes between SEVs and BEVs. In addition, several devices included in this analysis (ACURATE Neo, Evolut R, Portico) are no longer considered new-generation platforms, as updated versions with redesigned sealing skirts and frames are now available. This may partly explain the differences observed when compared to modern TAVR benchmarks. Finally, the relatively small number of patients receiving certain valve types (Allegra, Portico) limits the statistical power to detect differences across subgroups.

## Data Availability

The raw data supporting the conclusions of this article will be made available by the authors, without undue reservation.
